# Proteomics Based Identification of Proteins with Deregulated Expression in B Cell Lymphomas

**DOI:** 10.1371/journal.pone.0146624

**Published:** 2016-01-11

**Authors:** Rui Wu, Marcel Nijland, Bea Rutgers, Rianne Veenstra, Myra Langendonk, Lotte E. van der Meeren, Philip M. Kluin, Guanwu Li, Arjan Diepstra, Jen-Fu Chiu, Anke van den Berg, Lydia Visser

**Affiliations:** 1 Department of Pathology and Medical Biology, University of Groningen and University Medical Center Groningen, Groningen, The Netherlands; 2 Department of Biochemistry, Open laboratory for Tumor Molecular Biology, Shantou University Medical College, Shantou, China; 3 Department of Hematology, University of Groningen and University Medical Center Groningen, Groningen, The Netherlands; The University of North Carolina at Chapel Hill, UNITED STATES

## Abstract

Follicular lymphoma and diffuse large B cell lymphomas comprise the main entities of adult B cell malignancies. Although multiple disease driving gene aberrations have been identified by gene expression and genomic studies, only a few studies focused at the protein level. We applied 2 dimensional gel electrophoresis to compare seven GC B cell non Hodgkin lymphoma (NHL) cell lines with a lymphoblastoid cell line (LCL). An average of 130 spots were at least two folds different in intensity between NHL cell lines and the LCL. We selected approximately 38 protein spots per NHL cell line and linked them to 145 unique spots based on the location in the gel. 34 spots that were found altered in at least three NHL cell lines when compared to LCL, were submitted for LC-MS/MS. This resulted in 28 unique proteins, a substantial proportion of these proteins were involved in cell motility and cell metabolism. Loss of expression of B2M, and gain of expression of PRDX1 and PPIA was confirmed in the cell lines and primary lymphoma tissue. Moreover, inhibition of PPIA with cyclosporine A blocked cell growth of the cell lines, the effect size was associated with the PPIA expression levels. In conclusion, we identified multiple differentially expressed proteins by 2-D proteomics, and showed that some of these proteins might play a role in the pathogenesis of NHL.

## Introduction

Follicular lymphoma (FL) and diffuse large B cell lymphoma (DLBCL) compose 60% of non-Hodgkin lymphomas (NHLs), both are derived of germinal center or post germinal center B cells[1]. FL is usually an indolent lymphoma, while DLBCL is an aggressive lymphoma[2,3]. Transformation from FL to DLBCL occurs in 25–30% of the patients[4]. Gene expression profiling of DLBCL showed a distinct clustering of cases into two main groups, i.e. germinal center B cell like (GCB) and activated B cell like (ABC) DLBCL[5,6].

To study the transforming mechanisms for germinal B cell derived lymphomas at the protein level several proteomics based studies have been conducted. The follicular lymphoma derived cell line SUDHL-4 was used to identify secreted proteins[7]. In this study 209 proteins were found with a number of potential candidates for screening, diagnosis and monitoring of treatment efficiency[7]. Mixtures of cell lines were used to perform quantitative analyses by 2-D gel electrophoresis and SILAC approaches[8–10]. Fujii et al[8,9] compared 42 cell lines including Hodgkin lymphoma, B, T and NK cell lymphomas to a reference sample which was a mixture of all cell lines by quantative proteomics. The resulting expression profiles of 389 proteins were used to compare between the different groups of cell lines. Super SILAC was used to compare cell lysates of 5 GCB and 5 ABC DLBCL cell lines using a heavy stable isotype labelled mixture of cell lines as a reference. This yielded a proteome consisting of 7,500 proteins and a subset of 55 proteins that could differentiate between GCB and ABC DLBCL[10]. Comparison of normal B cells, LPS activated B cells and transgenic Eμ-driven murine B cell lymphoma by 2-D gel electrophoresis revealed 48 differentially expressed proteins[11].

In this study we compared the 2-D proteome profiles of NHL cell lines to Epstein Barr virus (EBV) transformed lymphoblastoid cell lines (LCL) to identify differentially expressed proteins. Expression of a selection of the differentially expressed proteins (B2M, PRDX1 and PPIA) was validated in the cell lines and in primary patient material. Inhibition of PPIA with cyclosporine A (CsA) showed a clear effect on cell growth in all NHL cell lines with a correlation between PPIA expression and sensitivity to CsA induced cell death.

## Materials and Methods

### Cell lines

DOHH2, SUDHL4 (FL), SUDHL6, SUDHL10, OCILY3, Karpas 422 and SUDHL5 (DLBCL) were obtained from DSMZ (Braunschweig, Germany). DOHH2, SUDHL4, OCILY3, Karpas 422 cells were routinely grown at 37°C at 5% CO_2_ in RPMI 1640 supplemented with 10% fetal calf serum (FCS), ultra-glutamine, penicillin and streptomycin (100U/ml). SUDHL5, SUDHL6 and SUDHL10 cells were cultured with 20% FCS. Five LCLs were generated from peripheral blood mononuclear cells by infection with B95.8 virus. One LCL was used to compare in the 2-D experiments, the other four were used in the validation and functional studies. LCLs were routinely grown in RPMI 1640 with 10% FCS. For the production of LCLs from peripheral blood permission was granted by the Institutional Review board (medical ethical committee UMCG) and written informed consent was obtained.

### Patient material

Tissue samples of 46 patients were collected from the pathology biobank for validation by immunohistochemistry. These 46 cases, consisted of 13 low grade FL, 8 FL transformed to DLBCL with evidence of FL in the sample or earlier diagnosis of FL (TFL), and 25 nodal DLBCL. The 25 DLBCL cases were stained for CD10, BCL6 and MUM1 and classified according to the Hans algorithm[12] in GCB (n = 14) and ABC (non-GCB, n = 11). A second group of 137 DLBCL NOS patients, of which 12 patients were also included in the first cohort, was used for the validation of B2M expression. The study protocol was consistent with international ethical and professional guidelines (the Declaration of Helsinki and the International Conference on Harmonization Guidelines for Good Clinical Practice). The use of anonymous rest material is regulated under the code for good clinical practice in the Netherlands. Informed consent was waived in accordance with Dutch regulations.

### Protein extraction

Cells (5–10 x 10^8^) were homogenized in 1 ml of the Homogenize Buffer Mix (BioVision, Milpitas, CA USA) in an ice-cold Dounce homogenizer. The homogenate was centrifuged at 700g for 10 minutes at 4°C. The supernatants were transferred to a new tube and centrifuged at 10,000g for 30 minutes at 4°C. The total cellular membrane protein pellet was lyzed in lysis buffer (8M urea, 4% CHAPS, 2% Pharmalyte) and kept on ice for 30 minutes. The supernatants were harvested by centrifuging at 16,000g for 5 minutes at 4°C. The protein concentrations of the lysates were determined by Bradford assay.

### Two-dimensional polyacrylamide gel electrophoresis fractionation of cell extracts

100 μg protein was admixed with rehydration buffer (8M urea, 2% CHAPS, 0.28% dithiothreitol and 0.5% Pharmalyte pH 3–10). Immobilized pH gradient strips (11 cm, pH 3–10) were rehydrated for 12–16 hours after the protein was loaded. Isoelectric focusing (Bio-Rad, Shanghai, China) was performed at 20°C by the following program: a linear increase from 0–500V over 30 minutes, 500–1000V over 1 hour, 1000–5000V over 4 hours, 5000–8000V 4 hour and then held at 8000V for a total of 64,000Vh. This was followed by a two-step equilibration; first, strips were put into 10 ml equilibration buffer (6M urea, 30% glycerol, 2% SDS, and 50mM Tris-HCl, pH 8.8), which contained 1% dithiothreitol for 15 minutes; next, strips were put into 10 ml equilibration buffer with 2.5% iodoacetamide for 25 minutes, and transferred to 12% SDS polyacrylamide gels. All proteins were visualized by silver staining of the gel, according to standard protocols.

In each experiment, two gels were run in parallel, one with the LCL sample and the second gel with one of the lymphoma cell lines. In order to assure reproducibility, all samples were run at least twice.

All gels were scanned with a GS-710 calibrated imaging densitometer imager. The comparative analysis of gels was performed with PD Quest software (BioRad). The density of each spot was evaluated by normalizing volumes of all spots. Spots which were consistently up or downregulated (≥ 2-fold) or spots that appeared or disappeared and were showing consistent differences between LCL and NHL were carefully cut out. For LC-MS/MS spots with the highest density were selected, destained and digested overnight with 5ng/μl trypsin (freshly made in 20mM ammonium bicarbonate pH 8–8.5). After incubation, formic acid was added and gels were incubated 5 minutes on a shaker. They were centrifuged at 5,000rpm for 1 minute and the supernatant was collected for LC-MS/MS analysis with the LTQ-Orbitrap XL (Thermo Scientific, Bremen, Germany).

### Protein identification

The peaks and sequences of peptides from selected protein spots were identified by ProteinPilot 3.0 (Applied Biosystems). Proteins were identified by using the UniprotKB/Swiss-Prot database[13]. Proteins with the correct molecular weight and the highest peptide coverage were considered as the correct protein.

### Flow cytometry

Cells were collected by centrifuging at 1200rpm for 5 minutes at 4°C and incubated with an anti-B2M antibody (1:750, Dako, Glostrup, Denmark) for 30 minutes on ice. Cells were washed with 1ml 1% PBS/BSA and FITC labeled goat anti rabbit antibody (1:10, Southern Biotech, Birmingham, AL USA) was added as the secondary antibody. Acquisition was performed on a Calibur flow cytometer (BD Biosciences, San Jose, CA USA) and data were analyzed with Winlist software.

### Quantitative RT-PCR

Total RNA was isolated using QIAzol (Carlsbad, CA USA) and samples were DNAse treated (Ambion, Foster City, CA USA) according to the manufacturer’s protocol for cell lines. RNA concentration was quantified using the Nanodrop^™^ 1000 Spectrophotometer (Thermo Fisher Scientific Inc., Waltham, MA USA) and RNA integrity was evaluated by 1% agarose electrophoresis. cDNA was synthesized using 500ng input RNA, Superscript II and random primer according to the manufacturer’s protocol (Invitrogen, Bleiswijk, the Netherlands). Primers used were for PRDX1 forward 5’-AGCCTGTCTGACTACAAAGGAAAATAT-3’ and reverse 5’- GGCACACAAAGGTGAAGTCAAG-3’ and for PPIA forward 5’- AGCTGTTTGCAGACAAGGTCC-3’ and reverse 5’-GCAGGAACCCTTATAACCAAATCC-3’. The qPCR reaction was performed in triplicate in a final volume of 10μl consisting of 5μl SYBR Green mix (Applied Biosystems, Foster City, CA USA), 2μl of forward and reverse primer (300mM) and 2,5μl 1ng of cDNA. Amplification was performed on a Roche LightCycler^®^ 480 Instrument (Roche, Almere, the Netherlands). TBP was used as a housekeeping gene and 2^-ΔCp^ values were calculated.

### Immunohistochemistry

Immunohistochemistry was performed according to standard protocols with appropriate positive and negative controls. Antibodies used were: anti-B2M (1:200, antigen retrieval with TRIS/EDTA pH9, Dako), anti-PRDX1 (1:200, antigen retrieval with citrate buffer pH6, Abcam, Cambridge, UK) and anti-PPIA (1:800, antigen retrieval with citrate buffer pH6, Abcam).

### Cytotoxicity assay

Cell lines were cultured in triplicate at 10^5^ cells/ml with different concentrations (0–10μg/ml) of Cyclosporine A and Alamar Blue (Abd Serotec, Oxford, UK). Cultures were measured every 24 hours for 3 days at an emission of 560nm and extinction of 590nm. Experiments were performed 3 times.

### Statistical analysis

Statistical analysis was performed with IBM SPSS Statistics 22. The Mann-Whitney U-test was used to compare B2M, PRDX1, and PPIA expression levels in NHL groups and LCLs for MFI and mRNA levels. Differences of B2M, PRDX1, and PPIA staining were defined by Chi-square test for immunohistochemistry. A paired T-test was performed to define the difference in cell viability before and after cyclosporine A treatment. The correlation of PPIA expression level and cell viability was defined by Spearman-test. All analyses were two-tailed. P<0.05 was considered as significant.

## Results

### Proteome profiles of LCL and NHL cell lines and protein identification

An average of 1,133 (±355) and 1,119 (±330) spots were detected in the 2-D gels of the LCL and NHL cell lines, respectively (Figure A in S1 File). The paired match rate of spots on control gels to lymphoma cell line gels ranged from 92 to 96% indicating a good consistency. We excluded spots that were too weak in both gels or that were incorrectly annotated. This resulted in an average of 248 (±24) reliable spots per gel that could be used for differential expression analysis. An average of 130 (±25) spots were at least 2-fold up or downregulated between the paired NHL and LCL cell lines. We picked 38 (±4) protein spots in each pair of gels, based on sufficiently high expression levels to analyse and reliable separation on the 2-D gel. Based on the position in the 2-D gels we were able to link them to 145 unique protein spots. Of these 34 spots were found in at least three NHL cell lines. Spots that were consistently up or downregulated (n = 22) were pooled for protein identification, whereas spots that were up in some and downregulated in other NHL cell lines (n = 12 spots, resulting in 2x 12 protein IDs) were analyzed separately. For the 34 spots differentially expressed in 3 or more cell lines a total of 46 analyses were performed. The results are summarized in Table 1. Of the 12 spots that were analyzed in duplicate and were upregulated in some and downregulated in other cell lines, 7 represented the same protein, while 5 represented different proteins with similar molecular weights. Of those 5 spots (10 different proteins) only 4 were found in at least 3 cell lines, the other 6 were removed from further analysis. Four proteins were found twice (PFN1, CFL1, PRDX1 and PPIA) at similar weight but at different iso-electric focusing points, probably due to posttranslational modifications such as phosphorylation, and are indicated as modified.

**Table 1:** Altered expression of proteins identified by LC-MS/MS.

spot number	protein name	UniProtKB/Swiss-Prot	Mass (kDa)	Sequence coverage (%)	Excluded from heatmap
1	RPSA	P08865	32.854	64	
2	TUBB	P07437	49.671	45	
3	PRDX1*	Q06830	22.11	76	
4	PRDX3	P30048	27.693	59	
5	CALR	P27797	48.142	67	
6	PRDX2	P32119	21.892	79	
7	MDH1	P40925	36.426	57	
8	ARPC5	O15511	16.32	77	
9	PFN1	P07737	15.054	74	
10	CALM1	P62158	16.838	48	
11	LYZ	P61626	16.537	18	
12	RPS12	P25398	14.515	76	
13	ATIC	P31939	64.616	66	
14	PCBP1	Q15365	37.498	67	
15	ENO1	P06733	47.169	47	
16A	PRDX4	Q13162	30.54	10	
16B	TPI1	P60174	30.791	60	
17A	EEF1B2	P24534	24.764	70	×
17B	NUDT5	Q9UKK9	24.328	19	×
18	CFL1	P23528	18.502	73	
19A	LGALS1	P09382	14.716	81	
19B	LGALS1	P09382	14.716	73	
20	PPIA*	P62937	18.012	75	
21A	PFN1*	P07737	15.054	69	
21B	PFN1*	P07737	15.054	84	
22A	ECHS1	P30084	31.387	72	×
22B	ACO2	Q99798	85.425	54	×
23A	GNB2L1	P63244	35.077	18	×
23B	MDH2	P40926	35.503	51	
24A	FAHD1	Q6P587	24.843	37	
24B	HSD17B10	Q99714	26.923	26	×
25	CFL1*	P23528	18.502	83	
26A	PKM	P14618	57.937	69	
26B	PKM	P14618	57.937	4	
27	B2M	P61769	13.715	71	
28A	MYL6	P60660	16.961	63	
28B	MYL6	P60660	16.961	26	
29	SSBP1	Q04837	17.260	27	
30A	CAPZA1	P572907	32.923	57	
30B	CAPZA1	P572907	32.923	26	
31A	GSTP1	P09211	23.356	32	
31B	GSTP1	P09211	23.356	68	
32A	IDH3A	P50213	39.592	27	
32B	IDH3A	P50213	39.592	29	
33	PRDX1	Q06830	22.110	53	
34	PPIA	P62937	18.012	82	

*: modified protein

**Fig 1:**
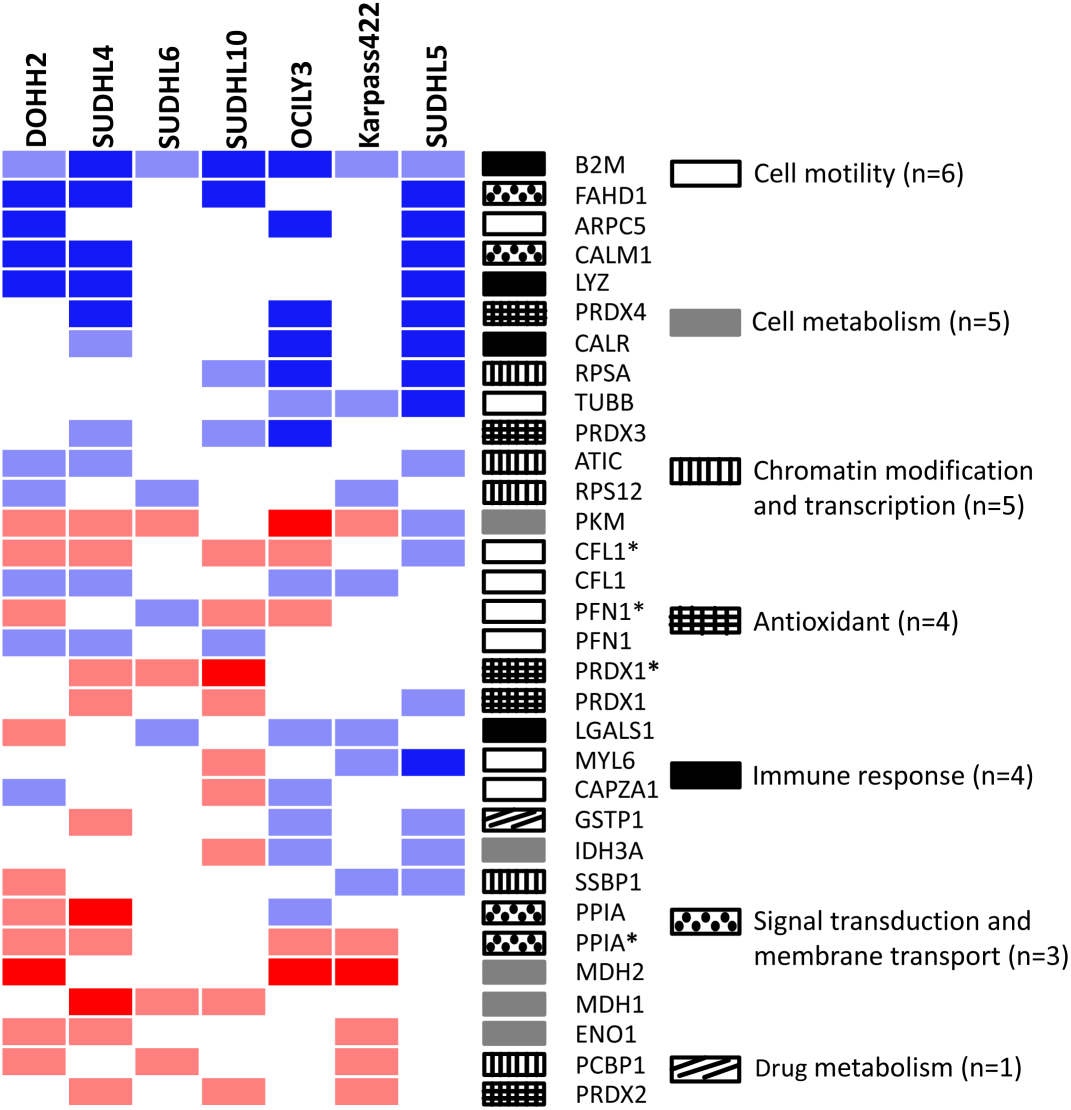
Heatmap of 2-D spots with different intensities in NHL compared with the LCL cell line. Proteins that were differentially expressed in at least 3 cell lines are shown. In total, 28 different proteins have been found and 4 proteins were also found in a modified form (*). Blue: protein spot is missing in NHL cell lines. Light blue: protein spot is down-regulated in NHL cell lines. Red: protein spot is missing in LCLs and thus increased in NHL cell lines. Light red: Protein spot is weaker in LCL and thus elevated in NHL cell lines. Gene ontology was checked to classify the proteins according to function.

Fourteen proteins, i.e. B2M, FAHD1, PRDX4, LYZ, CALM1, ARPC5, CALR, TUBB, PRDX3, RPSA, ATIC, RPS12, PFN1, and CFL1, were downregulated in NHL cell lines compared to LCL cell lines and 8 proteins, i.e. CFL1 (modified), PPIA (modified), MDH2, PRDX1 (modified), MDH1, ENO1, PRDX2 and PCBP1 were upregulated (Fig 1). The remaining 10 proteins, i.e. LGALS1, PFN1 (modified), MYL6, SSBP1, CAPZA1, GSTP1, IDH3A, PPIA, PRDX1 and PKM, were downregulated in some of the NHL cell lines and upregulated in others. The identified proteins are involved in cell motility (n = 6), cell metabolism (n = 5), chromatin modification and transcription (n = 5), anti-oxidant (n = 4), immune response (n = 4), signal transduction and membrane transport (n = 3) and drug metabolism (n = 1)(Fig 1).

We selected B2M, PRDX1 and PPIA for further validation based on the differential expression patterns in the NHL cell lines and availability of suitable antibodies for immunohistochemistry. B2M plays a role in the immune response, PRDX1 has an anti-oxidant function, and PPIA is involved in signal transduction.

### Validation of B2M

**Fig 2:**
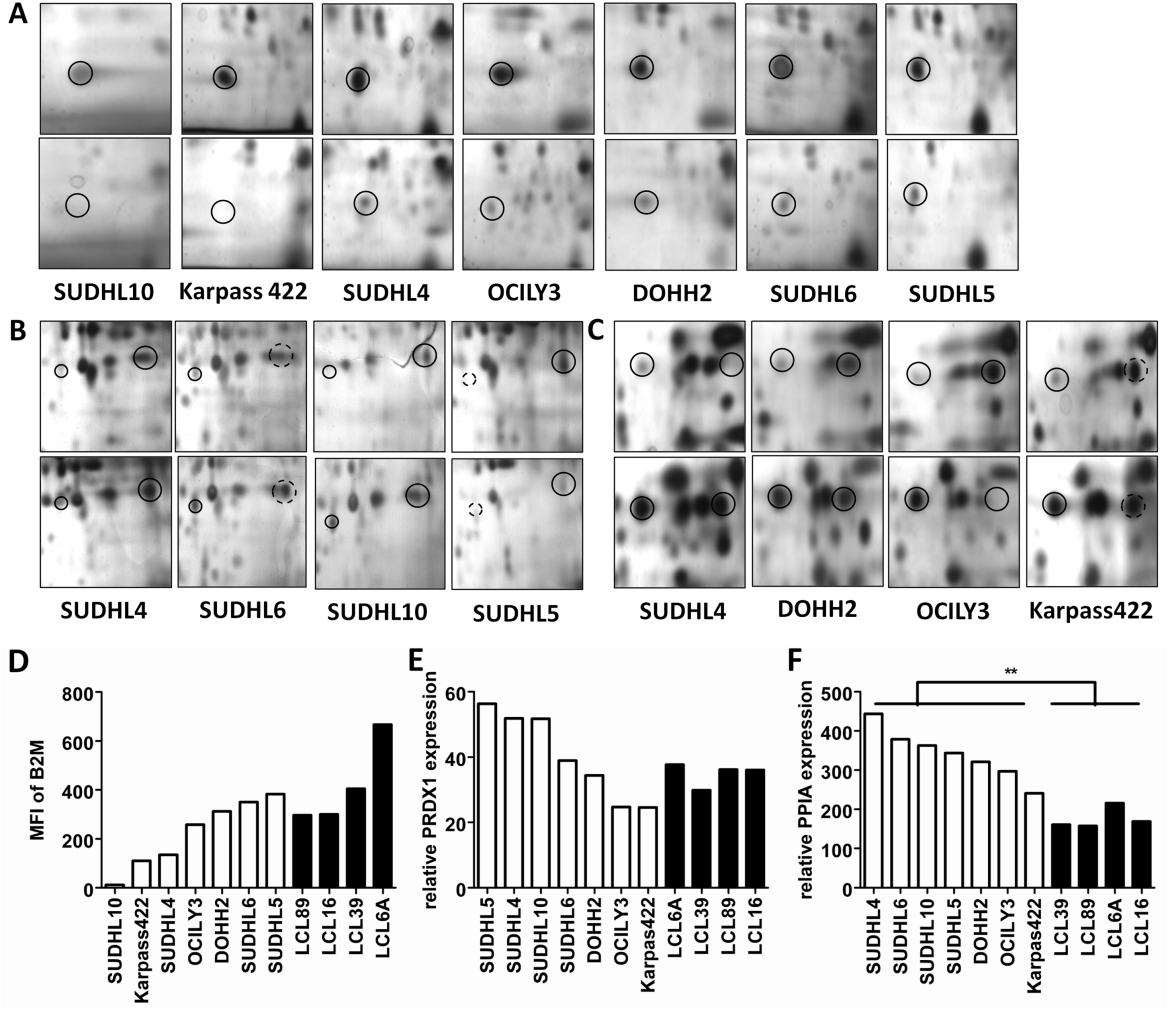
Expression of B2M, PRDX1 and PPIA. **(A-C)** Silver stained 2 Dimensional SDS PAGE gels. Differential expressed spots have been circled. Continuous circles indicate differential expression was more than 2 fold. Dotted circles indicate difference in expression level was less than 2 fold. Upper line pictures are LCL and lower lines are NHL cell lines. **(A)** SUDHL10, Karpas 422, SUDHL4, OCILY3, DOHH2, SUDHL6 and SUDHL5 are shown. Loss of B2M is observed in SUDHL10 and Karpas 422, decreased expression of B2M is seen in SUDHL4, OCILY3 and DOHH2, and moderate decreased expression of B2M is seen in SUDHL6 and SUDHL5 compared to LCL. **(B)** SUDHL4, SUDHL6, SUDHL10 and SUDHL5 are shown. Modified proteins are located on the left. Elevated expression of PRDX1 is observed in SUDHL4, SUDHL10 and decreased expression of PRDX1 in SUDHL5 compared to LCL. Elevated expression of modified PRDX1 is observed in SUDHL4, SUDHL10 and SUDHL5 compared to LCL. **(C)** SUDHL4, DOHH2, Karpas 422 and OCILY3 are shown. Modified proteins are located on the left. Elevated expression of PPIA is observed in SUDHL4, DOHH2 and decreased expression of PPIA in OCILY3 compared to LCL. Elevated expression of modified PPIA is observed in SUDHL4, DOHH2, OCILY3 and Karpas 422 compared to LCL. **(D)** The mean fluorescent intensity of B2M expression determined by flow cytometry. **(E)** The mRNA expression level of PRDX1. **(F)** The mRNA expression level of PPIA. Mann-Whitney U test was used to compare the differences in B2M, PRDX1, and PPIA expression between the NHL cell line group and LCL group (**: p<0.01).

**Fig 3:**
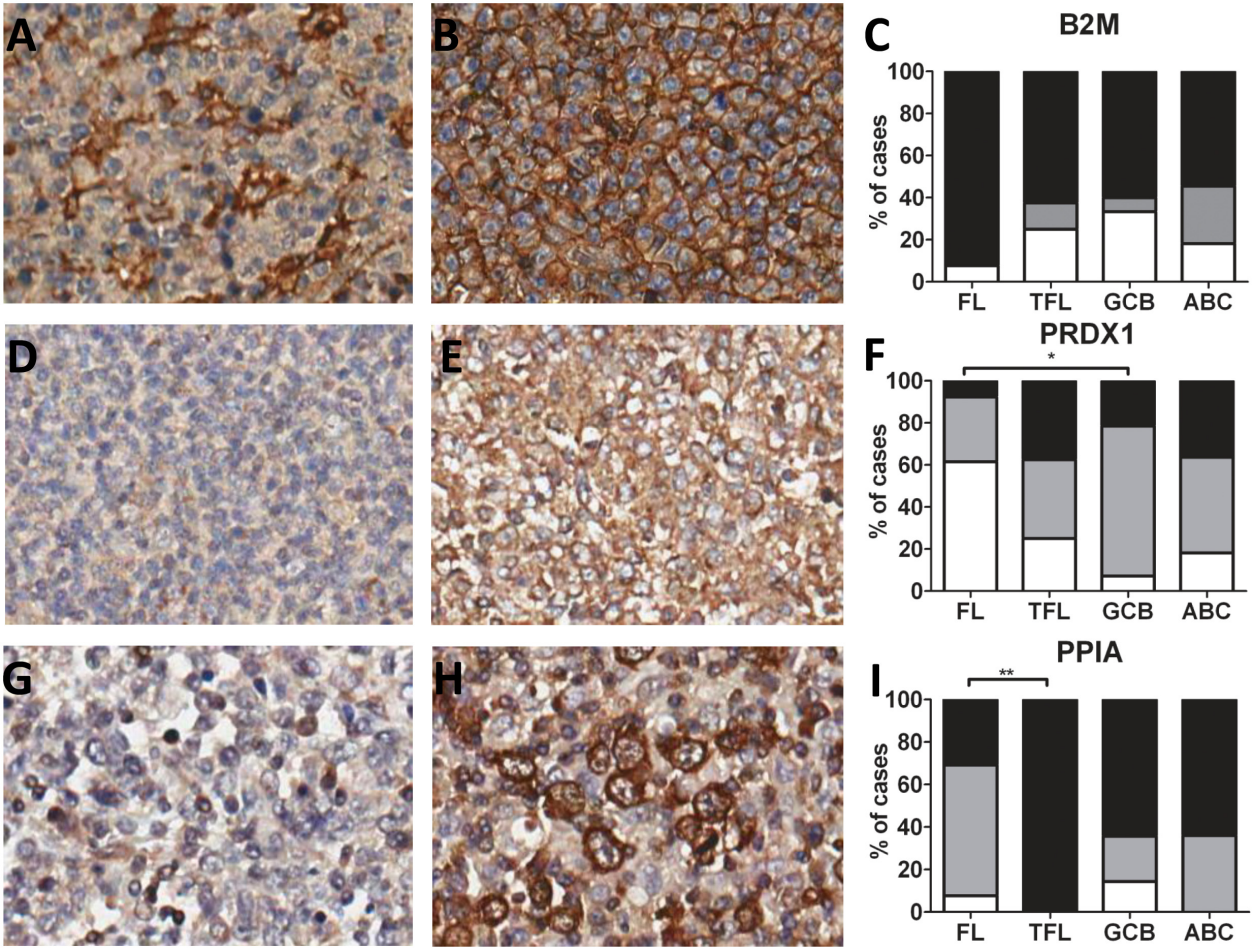
Immunohistochemistry staining of B2M, PRDX1 and PPIA. **(A-C)** B2M, **(D-F)** PRDX1, **(G-I)** PPIA. **(A, D, G)** negative cases, **(B, E, H)** positive cases. **(C, F, I)** Percentage of positive cases in different types of NHLs. Black bar indicates positive staining; grey bar indicates cytoplasmic staining for B2M and weak staining for PRDX1 and PPIA; white bar indicates negative staining. Chi-square test were performed to determine the differences in the B2M, PRDX1, and PPIA staining between the NHL subtypes (*: p<0.05; **: p< 0.01).

Expression of B2M was absent or reduced in all NHL cell lines compared to the LCL cell line in the 2-D analysis (Fig 2A). These 2-D results were validated on the cell lines by flow cytometry (Fig 2D). Four of the NHL cell lines showed lower mean fluorescent intensity (MFI) as compared to the 4 LCLs, consistent with the 2-D analysis, whereas the other three cell lines showed a similar MFI. Immunohistochemistry of 46 primary cases revealed total loss of B2M expression in 21% of cases, i.e. 1 out of 13 FL, 2 out of 8 TFL, 5 out of 14 GCB, and 2 out of 11 ABC. In addition, 9% of the patients showed cytoplasmic staining for B2M, i.e. 1 TFL, 1 GCB, 3 ABC (Fig 3A–3C), so in total 30% of cases showed no membrane expression of B2M. Since B2M loss was most common in DLBCL patients, we further checked loss of B2M expression in a larger cohort of 137 DLBCL patients. Of the 126 evaluable cases 35 (28%) were completely negative for B2M while 29 patients (23%) showed cytoplasmic expression of B2M, so a loss of membrane B2M expression was observed in a total of 51% of the DLBCL cases.

### Validation of PRDX1

PRDX1 (modified) was upregulated in 3 NHL cell lines (SUDHL4, SUDHL6 and SUDHL10) compared to the LCL cell line. Expression of PRDX1 was upregulated in 2 NHL cell lines (SUDHL4 and SUDHL10) and downregulated in 1 NHL cell line (SUDHL5)(Fig 2B). PRDX1 mRNA expression levels were higher in SUDHL4, SUDHL10 and SUDHL6 (Fig 2E) compared to the LCL cell lines, consistent with the 2-D results. In contrast, SUDHL5 had the highest mRNA levels, while in the 2-D experiment PRDX1 protein levels were downregulated compared to the LCL cell line.

Immunohistochemistry of the 46 primary NHL cases showed positive staining in 33 cases (72%). In FL, 5 of the 13 cases were positive with 4 cases showing weak positive and 1 case showing strong positive staining. Of the TFL cases, 3 showed weak positive staining and 3 strong staining. In GCB DLBCL 10 cases were weak positive and 3 cases were strong positive. Of the ABC DLBCL cases 5 showed weak positive staining and 4 showed strong staining (Fig 3D–3F). When comparing the staining pattern of PRDX1 in the NHL subtypes, a significant difference (p = 0.0409) was found. Comparison of the staining results between the three lymphoma subtypes revealed a significant difference between FL and GCB-DLBCL (p = 0.0112).

### Validation of PPIA

The expression of PPIA (modified) was upregulated in 4 cell lines (DOHH2, SUDHL4, OCILY3 and Karpas 422) compared to LCL in the 2-D gels. The unmodified PPIA was upregulated in 2 cell lines (DOHH2 and SUDHL4) and downregulated in 1 cell line (SUDHL6) (Fig 2C).

PPIA mRNA expression levels were upregulated in all NHL cell lines compared to LCLs (p = 0.0040, Fig 2F). SUDHL4 mRNA levels were highest, fitting the 2-D pattern, while the other NHL cell lines with upregulated protein levels in the 2-D (DOHH2, OCILY3 and Karpas 422) analysis were amongst the lowest at the mRNA levels. Immunohistochemistry of primary cases revealed in most of the FL cases (8 of 13) weak positive staining, while all 8 TFL cases showed strong positive staining for PPIA. GCB and ABC DLBCL staining results were comparable with 9 and 7 strong positive cases, for ABC the remaining cases were weak positive, while for GCB they were partly weak and partly negative. The results are summarized in Fig 3G–3I. The pattern of PPIA expression in primary patient material was significantly different (p = 0.0418) with the significant difference between FL and TFL (p = 0.0079).

**Fig 4:**
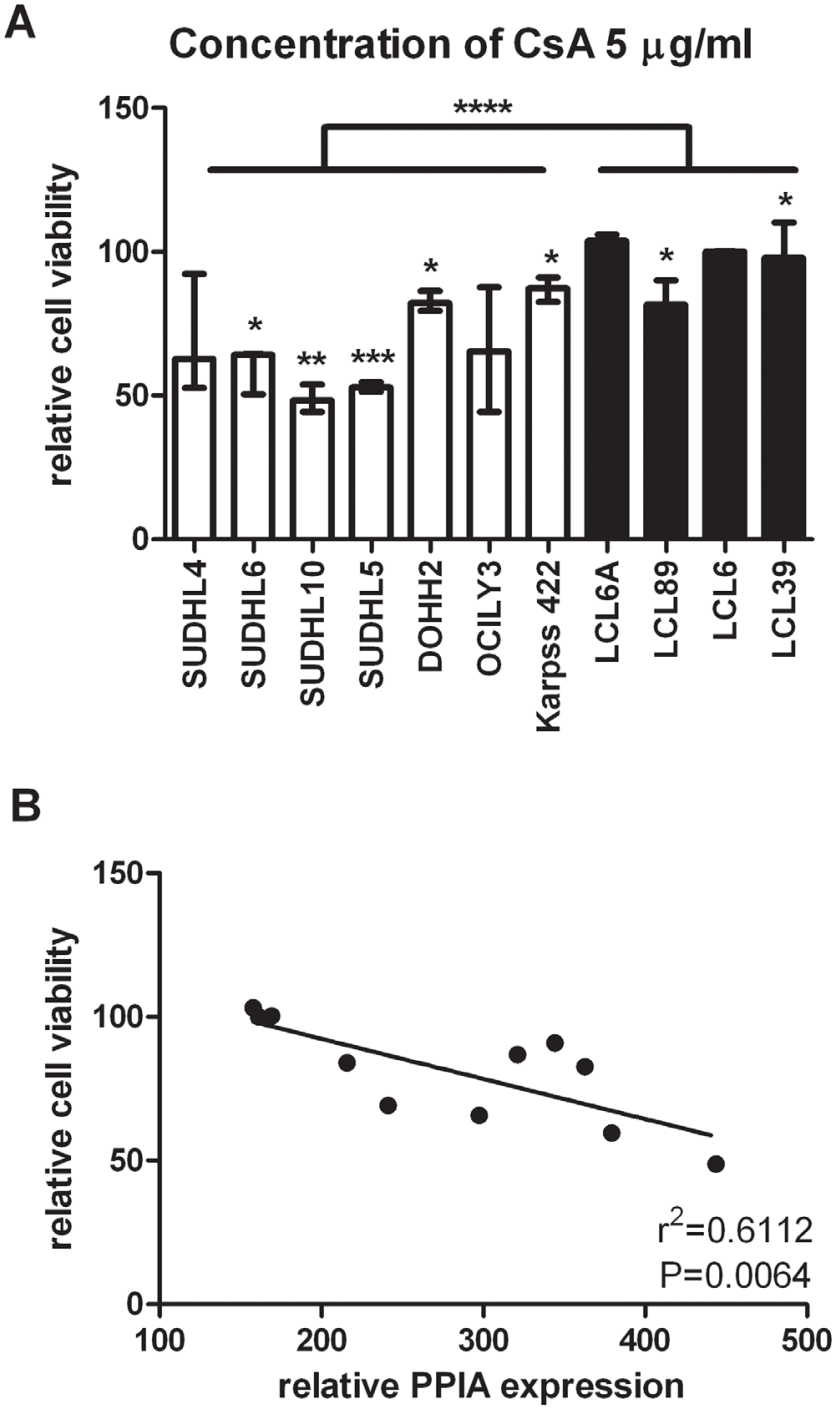
Sensitivity of NHL and LCL cell lines to inhibition of PPIA by 5 μg/ml CsA. **(A)** The inhibition of cell viability after CsA treatment. After adding 5 μg/ml CsA for 72 hours, the viability of NHL and LCL cell lines was evaluated by alamar blue assay. A paired T-test was performed to compare the cell viability before and after treatment. Mann-Whitney U test was used to compare the cell viability between the NHL cell line group and LCL group. (*: p<0.05; **: p<0.01; ***: p<0.001; ****: p<0.0001) **(B)** The correlation between PPIA expression level and the inhibition of cell viability after NHL cell lines treated with 5 μg/ml CsA for 72 hours. Statistical significance was determined by Spearman test (p = 0.0064, r^2^ = 0.6112). There is a negative correlation between the relative PPIA expression and the % of viability after CsA treatment.

To further investigate the role of PPIA in NHL, we inhibited PPIA in all cell lines with cyclosporine A (CsA). After treating the cells for 72 hours with different concentrations (0, 0.5, 1, 2, 5, 10 μg/ml) of CsA the viability of LCL and NHL cell lines was assessed (Fig 4; Figure B in S1 File). The NHL cell lines were significantly more sensitive to the effect of CsA than the LCL cell lines at each concentration. SUDHL5 and SUDHL10 were most sensitive. To explore whether the expression level of PPIA is related to the sensitivity of CsA treatment, we correlated the PPIA mRNA level with the relative cell viability of 7 NHL and 4 LCL cell lines upon treatment with CsA. A significant negative correlation was observed between PPIA mRNA level and the viability of cells after CsA treatment, with the most significant effect in cells treated with 5 μg/ml CsA (p = 0.0064, r^2^ = 0.6112; Fig 4; Figure B in S1 File).

## Discussion

Our 2-D gel electrophoresis approach revealed 28 differentially expressed proteins in lymphoma cell lines compared to LCLs. LCLs were chosen since they are cell lines and have similar proliferation patterns, while normal B cells are in a resting stage. For LCLs the transformation mechanism is by EBV and its proteins, and should be different from the NHL cell lines. The identified proteins are involved in various processes relevant to the pathogenesis of B cell lymphoma including cell motility and metabolism.

The proteins included in the cell motility gene ontology are ARPC5, TUBB, CFL1, MYL6, CAPZA1 and PFN1. With the exception of the modified CFL1 and PFN1, these proteins are all downregulated in NHL. ARPC5, CFL1, CAPZA1 and PFN1 have been shown to play roles in metastasis and invasion of solid tumors. Downregulation of ARPC5 blocks metastasis[14], whereas downregulation of CAPZA1[15] and PFN1[16] enhances metastasis and invasion or motility of cells. The presence of phosphorylated CFL1[17] is associated with metastasis, and phosphorylated PFN1[18] leads to enhanced angiogenesis via the upregulation of HIF1α. The functional consequences of downregulation of this group of proteins in NHL remains unknown. The proteins associated with metabolism are involved in the process known as the Warburg effect[19], which is a hallmark of cancer. PKM and ENO-1 are part of glycolysis, while IDH3, MDH1 and MDH2 are part of the Krebs cycle. Four of the five metabolism proteins are upregulated in NHL, except for IDH3 which was downregulated in 2 cell lines and upregulated in one. The PRDX proteins (anti oxidative proteins) are indirectly related to the Warburg effect, since reactive oxygen species levels are up as a result of the Warburg effect and the PRDX proteins can neutralize that effect. Upregulation of PRDX1 and PRDX2 fits with the upregulation of metabolism proteins, however, PRDX3 and PRDX4 were downregulated in NHL compared to LCL cell lines.

Various proteins identified by us as differentially expressed in NHL compared to LCL have been identified in B cell lymphoma previously. In the Eμ-driven mouse model Romesser et al found differential expression of ENO1, CALR, CFL1 and PPIA by comparing the proteome of activated B cells to B cell lymphoma[11] Consistent with their results we found downregulation of CALR and upregulation of the modified CFL1 protein and PPIA in lymphoma compared to LCL cells. The unmodified CFL1 protein was downregulated in our experiments, suggesting a shift towards the modified form of the CFL1 protein, which could indicate a more active form due to for example phosphorylation. PRDX1 expression was reported in a LCL cell line[20] and in DLBCL[21]. We found upregulation of PRDX1 in NHL compared to LCL. PRDX4 was shown to be upregulated in DLBCL[21], while we found downregulation in NHL compared to LCL. Protein levels of GSTP1 were reported to be high in 29% of DLBCL cases and low in all FL cases[22] Expression of GSTP1 has also been found with 2-D electrophoresis in a LCL cell line[23]. In our study, GSTP1 was downregulated in 2 cell lines and upregulated in one cell line. Loss of B2M has been described previously in DLBCL of testis and the central nervous system as well as in DLBCL NOS[24,25]. B2M levels were decreased in all seven NHL cell lines compared to LCL. LGALS1 expression has been reported in 7% of DLBCL[26]. In LCLs and EBV+ post-transplant lymphoproliferative disorders expression of LGALS1 has been reported upregulated[27]. We found downregulation in 3 cell lines and upregulation in 1 cell line, comparable to the reported data. Thus 9 of 28 proteins identified in this study have been reported in B cell lymphoma before and showed expression changes consistent with the literature.

B2M, expression is downregulated or absent in all NHL cell lines compared to LCL cells. This observation is partially supported by flow cytometry and IHC staining. B2M is one of the polypeptide chains of human leukocyte antigen class I (HLA class I), and both chains are essential for membrane expression of HLA class I. Loss of HLA class I expression provides an immune escape mechanism for tumor cells. A number of studies have shown loss of HLA class I in DLBCL patients, especially in DLBCL presenting at immune privileged sites[28]. Loss of B2M membrane expression has been published in up to 60% of DLBCL cases[25]. In our DLBCL cohort we found loss of B2M membrane expression in 51% of cases. Another differentially expressed protein we identified, CALR, has also been associated with antigen presentation[29]. CALR expression is decreased or lost in some NHL cell lines compared to LCLs. CALR is a chaperone molecule for HLA class I stability, as well as a chaperone for misfolded proteins[29]. Loss of CALR might be part of the immune escape mechanism or alternatively be a consequence of the loss of HLA class I expression.

PRDX1 expression is elevated at protein and mRNA level in part of NHL cell lines compared to LCL cells. In patient samples, we observed a higher proportion of strong positive and positive PRDX1 expression in the more aggressive TFL and DLBCL as compared to the indolent FL, this difference is most pronounced in the comparison between GCB-DLBCL and FL. Peroxiredoxins are thiol peroxidases, that play a role in maintaining the redox balance and have anti-apoptotic ability. PRDX1 is expressed in germinal center B cells and plasma cells and in germinal center derived B cell lymphomas and multiple myeloma[21]. The expression of PRDX1 in NHL could play a role in protection against apoptosis or be part of the Warburg effect.

PPIA, expression was elevated in the 2-D in 3 cell lines and mRNA levels are consistently higher in NHL cell lines compared to LCL cells. In addition, PPIA protein expression is higher in aggressive TFL and DLBCL as compared to the indolent FL. PPIA belongs to the immunophilin family, which catalyzes cis-trans isomerization during protein folding. Elevation of PPIA expression has been observed in some solid cancers, such as non-small cell lung carcinoma[30] and gastric cancer[31]. Knockdown of PPIA can inhibit growth of non-small cell lung carcinoma cells[32]. PPIA supports neoplastic cell proliferation, cell cycle progression and invasion, while protecting them from apoptosis, by activating signaling pathways, such as NF-κB [33], and ERK1/2 [34]. PPIA can also be secreted into the circulation to attract monocytes and stimulates monocytes to produce IL-6[35,36], and thereby create a pro-tumor microenvironment. Cyclosporin A (CsA), a widely used immune suppressive drug, binds to PPIA and inhibits its function. In T cells, CsA inhibits transcription factor NF-AT which is important in activation of T cells. In our study, there is a correlation between PPIA mRNA level and CsA induced cell death. The lymphoma cell lines showed significant more inhibition of cell growth than the LCLs, at all tested CsA levels. CsA suppressed tumor progression of squamous cell carcinoma and murine B cell lymphoma in a mouse model[37]. In mouse xenograft models for bladder cancer[38] and breast cancer[39] CsA treatment prevented tumor growth. The use of CsA in patients has been tested in angioblastic T cell lymphoma, with a response to therapy in 8 out of 12 patients[40]. These data support CsA treatment as a novel therapy in PPIA positive DLBCL. However, the induction of EBV+ lymphoproliferative disease is a severe and well known side effect of long-term immune suppression by CsA in the post-transplantation setting.

In summary, we identified 28 differentially expressed proteins and validated the expression in primary tissue samples for three selected proteins. The finding of B2M and PRDX1 confirmed validity of our approach as it has been shown previously in NHL. Elevated PPIA expression in lymphoma compared with LCL cells is novel and its oncogenic potential is supported by the inhibition of cell growth upon CsA treatment.

## Supporting Information

S1 File (DOCX)
