# Supplementary material for: Proteomics Based Identification of Proteins with Deregulated Expression in B Cell Lymphomas
Source: PLoS One. 2016 Jan 11;11(1):e0146624. doi: 10.1371/journal.pone.0146624 (PMC4708982; doi:10.1371/journal.pone.0146624)
Supplement: S1 File — (DOCX) [file pone.0146624.s001.docx]

**
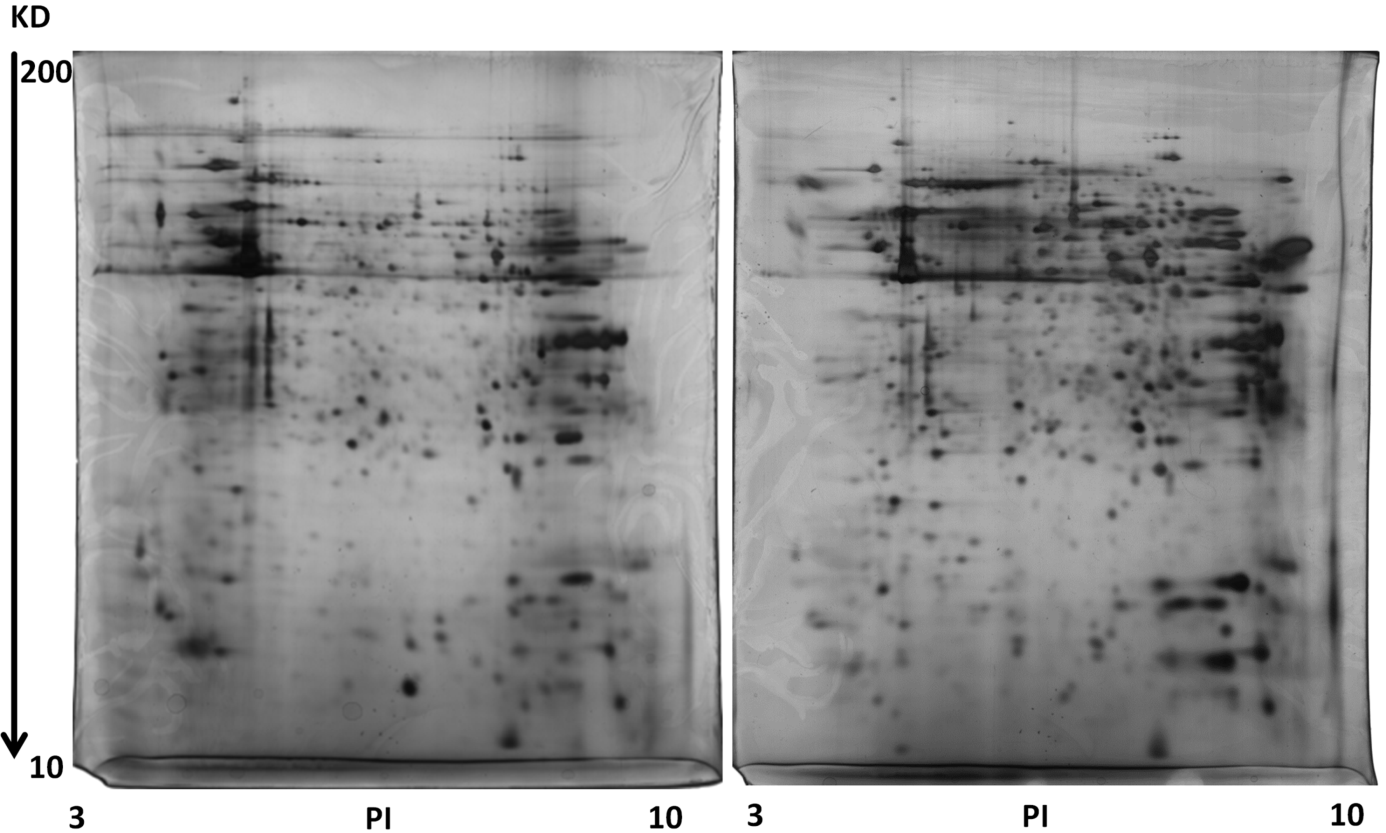
**

**S1 Fig A. Representative 2 dimensional gel of LCL and NHL cell line.** Silver stained 2 Dimensional SDS PAGE gels with the LCL cell line on the left and the SUDHL10 cell line on the right. PI ranges from 3 to 10 and molecular weight from 10 to 200 kDa. 1366 spots were found in the LCL cell line and 1300 in SUDHL10.

**
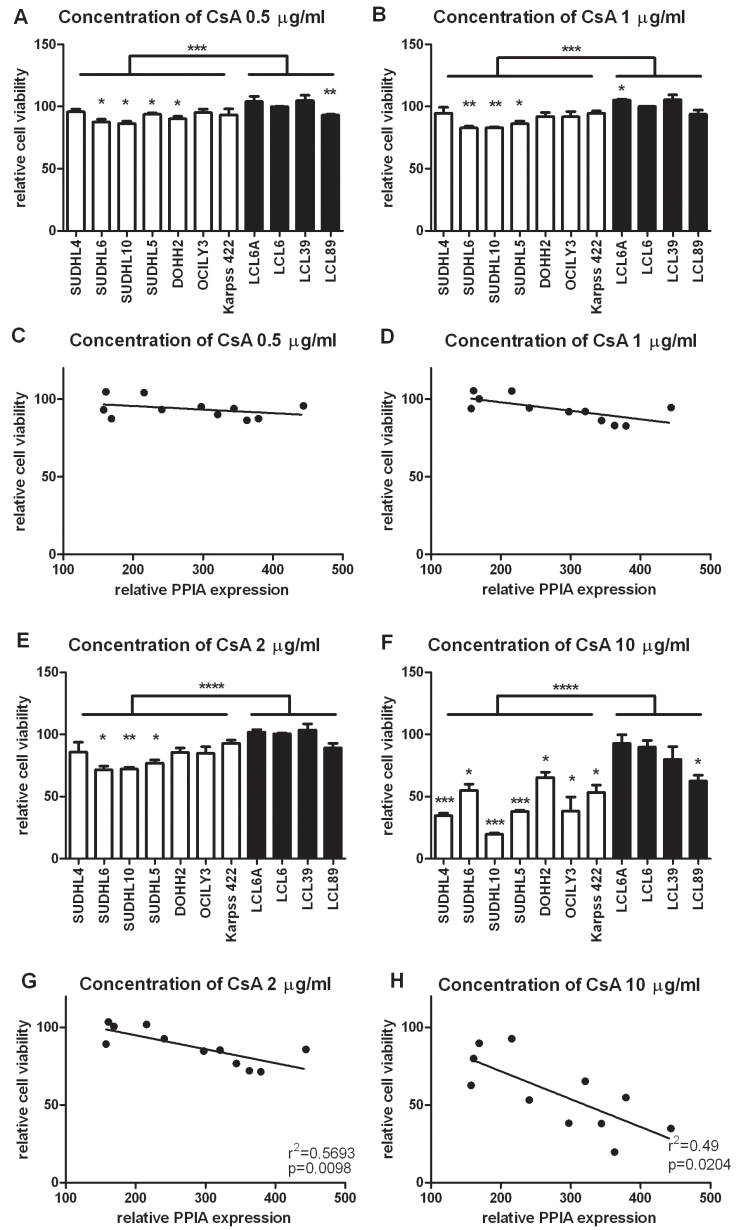
**

**S1 Fig B. Sensitivity of NHL and LCL cell lines to inhibition of PPIA by CsA at different concentrations.** **(A,B,E,F)** After adding different concentrations of CsA for 72 hours, the viability of 7 NHL and 4 LCL cell lines was evaluated by alamar blue assay. Paired- T test was performed to compare the cell viability before and after CsA treatment. Mann-Whitney U test was used to compared the cell viability between the NHL cell line group and LCL group. **(A)** 0.5 µg/ml CsA, **(B)** 1 µg/ml CsA, **(E)** 2 µg/ml CsA, **(F)** 10 µg/ml CsA. **(C, D, G, H)**The correlation between PPIA expression level and the inhibition of cell viability after NHL cell lines treated with CsA for 72 hours. Statistical significance was determined by Spearman test. **(C)** 0.5 µg/ml CsA, **(D)** 1 µg/ml CsA, **(G)** 2 µg/ml CsA, **(H)** 10 µg/ml CsA. (*: P<0.05; **: P<0.01; ***: P<0.001; ****: P< 0.0001)
